# Milk Intake at Midlife and Cognitive Decline over 20 Years. The Atherosclerosis Risk in Communities (ARIC) Study

**DOI:** 10.3390/nu9101134

**Published:** 2017-10-17

**Authors:** Natalia Petruski-Ivleva, Anna Kucharska-Newton, Priya Palta, David Couper, Katie Meyer, Misa Graff, Bernhard Haring, Richey Sharrett, Gerardo Heiss

**Affiliations:** 1 Department of Epidemiology, University of North Carolina, Chapel Hill, NC 27514, USA; anna_newton@unc.edu (A.K.-N.); palta@email.unc.edu (P.P.); migraff@email.unc.edu (M.G.); 2 Department of Biostatistics, University of North Carolina, Chapel Hill, NC 27514, USA; david_couper@unc.edu; 3 Department of Nutrition, University of North Carolina, Chapel Hill, NC 27514, USA; ktmeyer@email.unc.edu; 4 Department of Internal Medicine, Comprehensive Heart Failure Center, University of Wuerzburg, 97070 Würzburg, Germany; Haring_B@ukw.de; 5 Department of Epidemiology, Johns Hopkins Bloomberg School of Public Health, Baltimore, MD 21205, USA; rsharret@jhu.edu; 6 Department of Epidemiology, University of North Carolina, Chapel Hill, NC 27514, USA; gerardo_heiss@unc.edu

**Keywords:** lactose, lactase persistence, oxidative stress, cognitive decline, dementia, aging

## Abstract

**Background**: Faster rates of cognitive decline are likely to result in earlier onset of cognitive impairment and dementia. d-galactose, a derivative of lactose, is used in animal studies to induce neurodegeneration. Milk is the primary source of lactose in the human diet, and its effects on cognitive decline have not been fully evaluated. **Objective**: Assess the association of milk intake with change in cognitive function over 20 years. **Methods**: A total of 13,751 participants of the Atherosclerosis Risk in Communities (ARIC) cohort completed a food frequency questionnaire and three neurocognitive evaluations from 1990 through 2013. Two single nucleotide polymorphisms (SNPs) were used to determine lactase persistence (LCT-13910 C/T for Whites and LCT-14010 G/C for Blacks). Mixed-effects models were used to study the association of milk intake with cognitive change. Multiple imputations by chained equations were used to account for attrition. **Results**: Milk intake greater than 1 glass/day was associated with greater decline in the global z-score over a 20-year period. The difference in decline was 0.10 (95% CI: 0.16, 0.03) z-scores, or an additional 10% decline, relative to the group reporting “almost never” consuming milk. **Conclusions**: Replication of these results is warranted in diverse populations with greater milk intake and higher variability of lactase persistence genotype.

## 1. Introduction

Cognitive decline refers to the diminution in mental processes such as attention, short-term and long-term memory, reasoning, coordinating of movement, and planning of tasks, which are crucial for the conduct of daily living activities [1]. While the rate of decline in cognition varies among individuals [2,3,4,5], the factors affecting it are poorly understood, mainly due to limited long-term data on cognitive performance. Faster rates of decline may lead to earlier onset of cognitive impairment and dementia, resulting in significant burden to those affected and their caregivers [6,7]. Since evidence from neurobiological and cognitive performance studies suggest that age-related cognitive decline begins at midlife, the focus of research has shifted to modifiable risk factors and younger populations, to identify behaviors that can prevent or delay the progression to cognitive impairment [8]. 

Animal studies indicate that oxidative stress plays an important role in neurodegeneration [9,10,11,12]. The brain is particularly vulnerable to oxidative damage due to its high metabolic activity and low antioxidant defense [13,14,15,16,17,18,19,20,21,22,23]. Administration of d-galactose, a metabolic derivative of lactose, has been used extensively to mimic cognitive aging through oxidative stress in animal models [24,25,26,27,28,29]. d-galactose reacts readily with free amines of amino acids in proteins and peptides to form advanced glycation end products, which accumulate in the organs by binding with cell surface receptors or cross-linking with proteins, altering their structure and function and resulting in the generation of reactive oxygen species (ROS), increased oxidative stress, and inflammation [30,31,32,33,34]. Milk, the main source of lactose in the human diet, plays important roles in the growth and development of children due to its high fat and protein content, although its health effects in adults have not been studied as extensively [35,36,37]. In particular, few studies have explored the influence of milk on health outcomes by lactase persistent (LP) and non-persistent (LNP) genotype, which determines the pathways through which lactose in milk is metabolized [38,39]. In lactase persistence, lactose is broken down by the enzyme lactase in the small intestine, resulting in the formation of d-galactose—a contributor to ROS formation. Among those who are LNP, lactose is broken down in the colon by bacteria, resulting in the excessive formation of byproducts of bacterial fermentation, but not d-galactose. Since the two metabolic pathways differ significantly, the effect of lactose on health could differ by genotype. 

Studies looking at the association of milk intake with cognitive performance are few. Most are cross-sectional in design, have a small number of participants, or involve only older adults who had already experienced significant decline at the time of exposure assessment [40,41,42,43,44,45,46,47,48]. Thus, the aim of this study was to assess the association of milk intake in midlife with cognitive change over a 20-year period in a large biracial cohort, and to explore potential differences in the association by LP/LNP genotype. 

## 2. Materials and Methods 

### 2.1. Study Population

**Figure 1:**
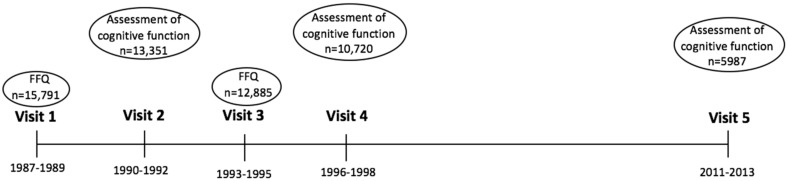
Timeline of the Atherosclerosis Risk in Communities (ARIC) study.

The Atherosclerosis Risk in Communities (ARIC) cohort is a prospective study of 15,792 adults who were selected through probability sampling from four US communities: Washington County, Maryland; Forsyth County, North Carolina; suburbs of Minneapolis, Minnesota; and Jackson, Mississippi. Participants were examined at five visits, with the first four visits approximately 3 years apart, and a fifth visit conducted 15 years following visit 4 (Figure 1). At baseline (1987–1989), the participants were 45–64 years of age, 56% were female, and 24% were Black. At the time of the study visits, participants received extensive examinations, including assessments of their medical conditions and physical functions. Annual (semi-annual since 2011) telephone follow-up interviews of ARIC cohort participants were also conducted [49]. A food frequency questionnaire (FFQ) was administered at visits 1 (1987–1989) and visit 3 (1993–1995). Cognitive function was assessed at visits 2 (1990–1992), 4 (1996–1998), and 5 (2011–2013). Analysis included participants who completed the FFQ on at least one occasion (visit 1) and those who completed cognitive assessments at visit 2, 4, and 5. Excluded were participants of race other than White or Black (*n* = 48) and Blacks from Washington County and Minneapolis (*n* = 55) due to small sample size. Also excluded were participants missing milk intake data (*n* = 27), those missing one or more cognitive function tests at baseline (*n* = 1649), and those with extreme reported caloric intake (<600 kcal or >4200 kcal per day for men, <500 kcal or >3600 kcal per day for women) (*n* = 261). 

### 2.2. Assessment of Cognitive Function

Verbal learning and short-term memory were assessed via the Delayed Word Recall Test (DWRT), in which participants were asked to learn 10 nouns, use them in sentences, and then recall those nouns after 5 min. The score on the test is the number of words recalled (0–10) [50]. Executive function was assessed via the Digit Symbol Substitution Test (DSST), during which participants use a key to write symbols corresponding to numbers in 90 s. The score on the test is the number of correctly written symbols from 0 to 93 [51]. Executive function and expressive language were assessed via the Word Fluency Test (WFT), during which participants generate as many words starting with the letters F, A, and S as possible within 60 s, with one trial per letter. The score on the test is the sum of all the correct words generated [52]. 

All test scores were converted to z-scores standardized to the visit 2 mean and standard deviation, calculated for each test by subtracting each participant’s test score at each visit from the visit 2 mean and dividing by the visit 2 standard deviation. Global cognition z-scores standardized to visit 2 global z mean and standard deviation were generated for each visit by averaging the z-scores of the 3 tests, and then subtracting the global mean and dividing by standard deviation from the visit 2 global z-score [53,54,55,56]. 

### 2.3. Assessment of Milk Intake 

An interviewer-administered food frequency questionnaire (FFQ) was used to assess dietary intake [57]. Total milk intake was estimated as combined intake of skim/low-fat and whole milk, reported in 8-ounce glasses with frequency of intake ranging from “Almost never” to “>6 times per day” in 9 categories. A number was assigned at mid-category of reported frequency (e.g., “3–5 times per day” = 4 times per day) for each type of milk to obtain the average daily intake in glasses/day, then added together across each milk type to obtain total milk intake, which was then reclassified into 4 categories: “Almost never”, “<1 glass/day”, “1 glass/day”, and “>1 glass/day”. Intake of all dairy included skim/low-fat and whole milk, yogurt, ice-cream, cottage cheese, other cheese, and butter in servings per day. One serving of dairy was equal to an 8-ounce cup of milk, 1 cup of yogurt, ½ cup of ice-cream, ½ cup cottage cheese, 1 slice of hard cheese, or 1 pat of butter. For participants with two FFQ assessments, an average was taken across visits for all dietary intake variables. For those with an FFQ at baseline only, the baseline reported amount was used.

### 2.4. Diet Quality Score

The Healthy Food Score, adapted from Steffen et al. [58,59] was created by summing the scores of food groups. Food groups included dairy other than milk (cottage cheese, other cheese, yogurt, ice cream, butter), vegetables, fruit (without juice), fruit juice, legumes, refined grain, whole grain, nuts, fish, meat (combined poultry, processed meat, beef, pork, and lamb), diet beverages, sugar-sweetened beverages, and coffee and tea. Daily intake of food groups was categorized into quintiles, except alcohol intake, legume, and beverages. Each quintile of food group intake was assigned a score: 0–4. For dairy, vegetables, fruit (without juice), fruit juice, refined grain, whole grain, nuts, and fish, scores were assigned in the following order: Quintile 1 = 0, Quintile 2 = 1, Quintile 3 = 2, Quintile 4 = 3, Quintile 5 = 4; for meat, the score was the reverse. Due to the limited range of intake, scoring for intake of legumes was 0, 1, and 2, if daily intake was 0, <1, and ≥1 serving, respectively. The score was reversed for diet beverages and sugar-sweetened beverages: 2, 1, and 0 for 0, >0 to <1, and one or more servings usually consumed per day, respectively. Daily coffee and tea intake was scored in five categories from 0 to 4, for 0, >0 to ≤2, >2 to ≤4, >4 to ≤6, and >6 cups per day, respectively. For alcohol intake, a score of 4 was assigned to the men who consumed between 10 and 50 g per day and to women who consumed between 5 and 30 g per day; otherwise a score of 0 was assigned [59].

### 2.5. Covariates

Analyses included the following covariates: visit 1 reported sex, race, study center, educational attainment (<high school, high school, >high school), time spent in moderate to vigorous physical activity in MET-minutes/week; visit 2 age, body mass index (BMI) in kg/m^2^, smoking status (ever smoker vs never smoker), alcohol consumption (ever drinker vs never drinker), diet quality score derived from the average of reported dietary intake [58,59]; visit 2 prevalent health condition such as diabetes, hypertension, coronary heart disease (CHD), and cancer. Diabetes was defined as fasting blood glucose level of ≥126 mg/dL, or non-fasting blood glucose level of ≥200 mg/dL, history of past diagnosis of diabetes by a physician, or diabetes medication use in the past 2 weeks. Hypertension was defined as diastolic blood pressure of ≥90 mm/Hg or systolic blood pressure of ≥140 measure at visit 2, or use of hypertension medication in the past 2 weeks. Prevalent CHD was defined as self-reported history of CHD at the baseline visit 1 or adjudicated CHD event between baseline and visit 2. CHD events included non-fatal myocardial infarction, coronary artery bypass surgery, or angioplasty. Prevalent cancer cases were defined as self-reported history of any cancer. Apolipoprotein E ε4 allele number (APOEe4) was included in analyses as it is a strong predictor of cognitive decline and risk for cognitive impairment.

### 2.6. Lactase Persistence Genotype

Lactase persistence, or the ability to digest lactose into glucose and galactose in adulthood, emerged 7500–10,000 years ago among populations that domesticated milk animals and consumed milk [60,61]. Dominant mutations occurred in the lactase promoter region upstream from the lactase phlorizin hydrolase locus on chromosome 2q21, retaining intestinal lactase into adulthood. The single nucleotide polymorphisms (SNPs) most frequently used to determine LP/LNP status are rs4988235 (LCT-13910C>T) in the populations of European descent and rs145946881 (LCT-14010G>C) in populations of African descent; however, studies in African countries suggest that there are other SNPs also associated with lactose digestion in these populations. The imputed genotypes LCT-13910 C/T in Whites [60] and LCT-14010G/C in Blacks [62] were used to denote LP/LNP in this cohort. Individuals with two minor alleles were classified as LNP.

Data on LP/LNP genotype were obtained for consenting ARIC participants using the Affymetrix Genome-Wide Human SNP Array 6.0 (Affymetrix, Santa Clara, CA, USA) and the IBC Chip Array (Affymetrix, Santa Clara, CA, USA). Genotypes were excluded for call rates <90%, MAF (minor allele frequency) <1%, Hardy–Weinberg equilibrium deviation <10^−6^, and genotype frequency that was different at *p* <10^−6^ from prior genotyped samples. Imputation was performed in two steps: (1) pre-phasing with ShapeIt, and (2) imputation with IMPUTE2. After frequency and genotyping pruning, there were 695,783 SNPs in the final set used for the imputation (669,450 autosomal SNPs). Final imputations were performed using IMPUTE2 based on the 1000 Genomes Phase I integrated variant set release (v3) in NCBI build 37 (hg19) reference panel haplotypes. All 1092 individuals were used for the imputation from the reference panel. The final sample with genetic data used for imputation was 9713 Whites and 2871 Blacks. Principal components were generated using the Eigensoft package (http://genepath.med.harvard.edu/~reich/Software.htm), and ancestry outliers were removed. The final sample with genetic data used for imputation was 9713 Whites and 2871 Blacks. 

### 2.7. Statistical Analysis

Baseline (visit 2) characteristics of the study population were reported by milk-intake category. To study the association between the four levels of milk intake and cognitive change from visit 2 to visit 5, we used mixed-effect models to account for repeated measures across study visits. A linear spline term was applied with a knot at six years, equal to the mean duration between visits 2 and 4 [53]. We performed the analyses using 3 models: (1) demographic model, adjusted for age, gender, and race-center; (2) full model, adjusted for age, gender, race-center, education level, APOEe4, BMI (kg/m^2^), smoking, drinking, diabetes, hypertension, physical activity (MET-min/week), total energy intake (kcal), and diet quality score; and (3) full model with food group replacing diet quality score (food groups that were significant in the model: protein (g/day), fat (g/day), servings of fruit, servings of vegetable, servings of sugar-sweetened beverages, and servings of non-milk dairy products for the association with total milk and skim/low-fat milk).

Analyses were stratified by race and by LP/LNP genotype. We used interaction terms with smoking, diabetes, diet quality score, fruit and vegetable intake, total fat intake, and physical activity to test for effect modification. Those variables were selected because of the previously reported association with cognitive performance or oxidative stress, the proposed mechanism through which milk intake could affect cognition. 

Attrition was addressed with multiple imputations by the chained equations (MICE) [63] method. The missing values for global z-score were imputed based on the observed values for a given individual, as well as the relations observed in the data for other participants. The values were imputed multiple times, creating a more accurate estimation of a standard error. Variables used to impute global z-scores and individual test scores for participants who did not attend visit 5, but were alive at the time, included retrospective ascertainment of hospitalization with dementia codes, Telephone Interview for Cognitive Status (TICS-m) questionnaire, clinical dementia rating (CDR) scale conducted with proxies, suspect dementia status, global z-scores from visit 2 and 4, as well as APOE4, demographic and socioeconomic (age, gender, race-center, BMI, education, income), behavioral (smoking and alcohol consumption), and cardiovascular risk factors (CHD, diabetes, hypertension, stroke, self-reported poor health). Interaction terms were derived empirically. Validation of the MICE approach for cognitive data in ARIC has been previously reported and it has been determined that MICE produced unbiased imputed values [64]. All statistical analyses were performed using Stata14.2 (StataCorp, College Station, TX, USA).

## 3. Results

### 3.1. Total Milk Intake

The final analytic set included 13,752 participants who had milk intake data and baseline cognitive performance data. Most participants (88%) reported milk intake on at least two occasions. The Pearson correlation coefficient for milk intake reported on two occasions was 0.44, which is consistent with previously reported estimates [65]. Average milk intake in this population was 0.87 glasses/day. Skim milk accounted for 75% of total milk intake. Overall, 11% of participants reported almost never drinking milk, 50% reported consuming <1 glass per day, 15% reported consuming 1 glass/day and 24% reported consuming >1 glass per day. A greater proportion of Black participants reported almost never drinking milk (16.2%, compared to 9.8% among Whites). Participant characteristics by milk intake group are presented in Table 1. Participants who reported drinking more milk were more likely to be male, White, have more years of education, have better diet quality score with greater intake of fruits and vegetables, have lower intake of meat and sugar-sweetened beverages (Table S1), and more time spent in moderate to vigorous physical activity. Baseline scores for the three cognitive tests did not differ by milk intake group (Table 1). 

**Figure 2:**
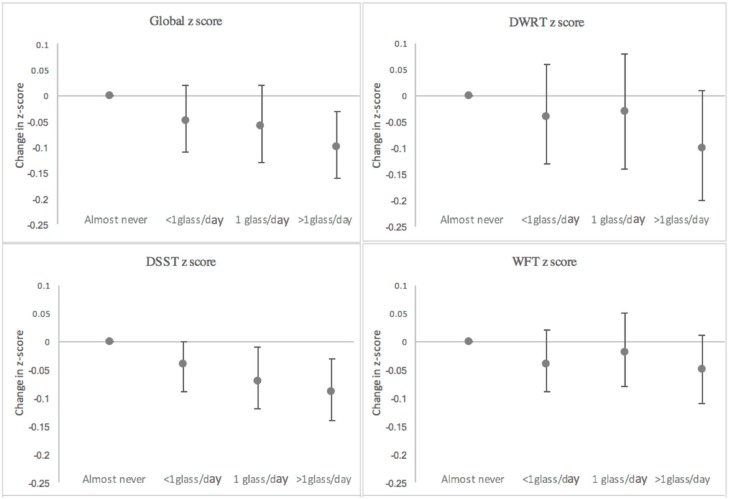
Estimated difference in the 20-year change in cognitive performance by milk intake group relative to those who reported “almost never” consuming milk adjusted for age, gender, race-center, education level, APOE4, BMI, smoking, alcohol intake, diabetes, physical activity, total energy intake and diet quality score. ARIC Study. Abbreviations: DWRT, delayed word recall test; DSST, digit symbol substitution test; WFT, word fluency test. Global z is a summary score, equal to the average of the three domain-specific z-scores.

Results of mixed model analyses suggest the presence of an association of milk intake with cognitive decline over a 20-year period (Table 2, Figure 2). The response was graded across milk intake categories. The difference in the 20-year change in global z-score between those who reported almost never drinking milk and those who reported drinking >1 glass/day was −0.10 (95% CI: −0.16, −0.03) z-scores, equivalent to a 10% additional decline. Decline in the DSST z-score (a test of processing speed) and DWRT z-score (a test of short-term memory) contributed the most to the difference in decline. 

**Figure 3:**
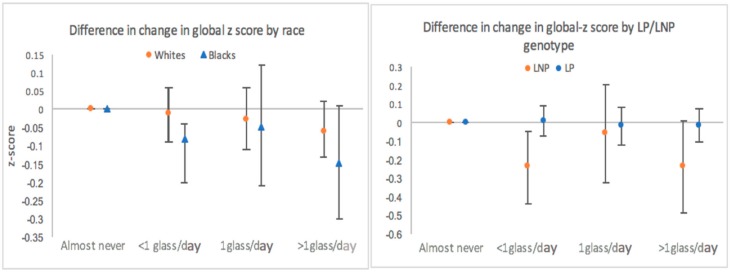
Estimated difference in the 20-year change in global z-score stratified by race and by LP/LNP genotype among Whites adjusted for age, gender, race-center, education level, APOE4, BMI, smoking, alcohol intake, diabetes, physical activity, total energy intake and diet quality. ARIC Study. Abbreviations: LNP, lactase non-persistence; LP, lactase persistence. “Almost never” used as a referent category.

We observed no effect modification of this association by race (Figure 3), or by other a priori hypothesized covariates (smoking, diabetes, diet quality score, fruit and vegetable intake, total fat intake, and physical activity). 

Availability of three cognitive assessments allowed us to compare change in cognitive function during two time periods: from visit 2 to visit 4 (6 years) and from visit 4 to visit 5 (14 years). Decline in cognitive function occurred at a faster rate during the later time period, however the difference in decline by milk intake group was observed during both times (Table S2). 

Estimates did not change when replacing diet quality score with individual food groups in the model (Model 2 vs. Model 3). 

### 3.2. Lactase Persistence

Among Whites, 9% of participants were classified as being lactase non-persistent. The presence of minor allele among Blacks was only 0.7%, with no participants being classified as lactase non-persistent. Thus, stratified analysis by LP/LNP genotype was restricted to Whites. Those who were classified as LP consumed on average more milk than those who were classified as LNP. Stratified analysis suggested that milk consumption may have a greater effect among those classified as LNP, however a graded response by milk intake group was not observed, possibly due to small numbers of participants classified as LNP (Figure 3). 

### 3.3. Skim/Low-Fat Milk and Total Dairy

The majority of participants reported drinking skim/low-fat milk, which accounted for 75% of total milk intake. Those who reported drinking more total milk also reported consuming more other dairy products and thus had greater all-dairy consumption overall (Table S3). Only 39 participants reported never consuming any dairy products, thus the exposure to all dairy products was classified into quartiles (Table S4). 

The association of skim/low-fat milk and all dairy with change in cognitive function was similar to the association observed with total milk. Those consuming more than 1 glass/day of skim/low-fat milk and those in the 4th quartile of all dairy intake experienced a faster rate of cognitive decline over the 20-year period. This was true for the overall population and in race-stratified analyses (Table S5). 

## 4. Discussion

This is one of the few prospective studies to examine the association of milk intake with cognitive performance. It is the only study of this association with multiple measures of cognitive function, allowing the assessment of change in cognition over time. 

Our results suggest that greater milk intake at midlife may be associated with greater rate of cognitive decline over a 20-year period. These results are consistent with results from a recent study of 3076 participants 65.5 years of age at the time of neurocognitive evaluation, in which milk consumption was associated negatively with verbal and working memory performance [48]. Three other prospective studies reported that full-fat milk intake was associated with poor cognitive function, and that high saturated fat intake from milk products was associated with poor cognitive function and increased risk of mild cognitive impairment [45,46,66], although the effect of fat from milk was emphasized, as opposed to lactose. 

We hypothesized that the effect of milk on cognitive function is through the effect of lactose on oxidative stress. Given the proposed mechanism, we chose total milk intake as the main exposure, since milk contains several times more lactose than any other dairy product, although associations of skim/low-fat milk and all dairy with cognitive decline were considered as part of sensitivity analyses, which showed similar associations.

After accounting for total fat intake in our model, the association of total milk, skim/low-fat, and total dairy with cognitive change remained constant. Further, there was no effect modification of the associations by tertiles of total fat intake, suggesting that the dairy fat content may not be the culprit in the observed faster rate of cognitive decline. 

The distribution of LP/LNP genotype in our population differed from the previously reported estimate in the US [67]. Only 9% of Whites where classified as LNP (as compared to previously reported 20%), and the SNP for LP/LNP among Blacks available in our study showed almost no variation. Considering that the estimated prevalence of LNP among Blacks in the US has been estimated at 80%, we concluded that the imputed SNP available in ARIC most likely did not characterize lactase persistence among Blacks [67,68].

The effect of milk intake on cognitive decline was greater among those participants classified as LNP. In the milk intake group with most participants of LNP genotype (<1 glass/day), we observed a significantly faster rate of decline over the 20-year period, compared to LNP participants who reported “almost never” drinking milk. However, due to the small number of participants characterized as LNP, we lacked power to capture the graded response that was observed in the overall analysis. Overall, findings from the LP/LNP stratified analysis did not support our hypothesis of milk intake having an effect on cognitive decline through the mechanism of d-galactose, which would have resulted in a greater decline among LP population. 

Our study had several limitations, including attrition, which is a concern for all longitudinal studies with long follow-up. Although attrition was addressed through MICE, taking into account a wide range of attributes influencing attrition, it is possible that we were not able to fully account for the effect of selective drop-out. Sensitivity analyses in which estimates of the rates of cognitive decline were adjusted for attrition and competing risk of death through inverse probability of attrition weighting yielded similar effect estimates (results not shown). Another limitation is the assumption that the assessment of average milk intake at visit 1 and visit 3 reflected long-term habitual intake throughout adulthood, which would have preceded the significant cognitive decline. Since diets change over the life course, exposure may have been misclassified for some individuals. Despite such limitations, the FFQ has been determined as a reliable method of assessing long-term intake and it is likely that the ranking of individuals with respect to milk intake was accurate [69]. In addition, we had two assessments of milk intake for most participants, thus we were able to reduce reporting error by taking the average across visits. 

Strengths of our study include a population-based biracial cohort of large size and with extensive follow-up, repeat assessments of the exposure and outcome, and data on three cognitive tests that permit a study of the association of milk intake with three cognitive domains. Assessment of exposure prior to the assessment of outcome reduced the likelihood for reverse causation, as poor cognitive health may affect dietary choices and ability to follow dietary recommendations and accurately report diet. Multiple assessments of cognitive function allowed capturing change in cognitive performance over time, which reduced confounding that is common to studies using one point in time assessment of cognitive performance [54]. 

## 5. Conclusions

Results of our study suggest that milk intake at midlife may be associated with a greater rate of cognitive decline from midlife to late-life. Further longitudinal studies in multiethnic groups, including those with higher prevalence of lactase non-persistence, are needed to better understand the link between milk intake and change in cognitive performance among adults. Other potential mechanisms through which milk intake may affect the rate cognitive decline should be explored.

## Figures and Tables

**Table 1:** Baseline (visit 2) characteristics of study participants by milk intake group. ARIC Study, 1990–1992.

Covariates	Milk Intake Group			
	Almost Never	<1 Glass/Day	1 Glass/Day	>1 Glass/Day
	*n* = 1554	*n* = 6872	*n* = 2036	*n* = 3290
Age, mean (SD)	56.7 (5.6)	57.2 (5.6)	58.5 (5.7)	57.9 (5.8)
Black, %	530 (34.1%)	1833 (26.7%)	360 (17.7%)	542 (16.5%)
Female, %	1023 (65.8%)	3879 (56.4%)	1096 (53.8%)	1664 (50.6%)
Study site, %				
Forsyth County, NC	328 (21.1%)	1894 (27.6%)	584 (28.7%)	760 (23.1%)
Jackson, MS	484 (31.1%)	1641 (23.9%)	320 (15.7%)	469 (14.3%)
Minneapolis, MN	335 (21.6%)	1499 (21.8%)	601 (29.5%)	1293 (39.3%)
Washington County, MD	407 (26.2%)	1838 (26.7%)	531 (26.1%)	768 (23.3%)
Education, % <High School	415 (26.8%)	1474 (21.5%)	390 (19.2%)	627 (19.1%)
Smoking, % Never	564 (36.3%)	2771 (40.3%)	839 (41.2%)	1301 (39.6%)
Drinking, % Never	366 (23.6%)	1582 (23.0%)	476 (23.4%)	654 (19.9%)
BMI (kg/m^2^), mean (SD)	27.9 (5.7)	28.1 (5.5)	27.7 (5.1)	27.9 (5.1)
Diabetes, %	220 (14.3%)	945 (13.8%)	311 (15.3%)	555 (16.9%)
Hypertension, %	622 (40.2%)	2451 (35.7%)	721 (35.6%)	1076 (32.8%)
Diet score, mean (SD)	19.3 (4.9)	20.7 (4.7)	22.0 (4.7)	22.1(4.8)
Lactose intake (g), mean (SD)	2.3 (3.0)	7.7 (5.5)	14.9 (3.5)	27.8 (15.4)
Physical activity (met-min/week)	500 (647)	674 (825)	822 (907)	728 (782)
APOEe4 allele, % present	565 (33.8%)	2218 (30.2%)	669 (30.7%)	1071 (30.3%)
Lactase persistence (Whites)				
CC (Lactase non-persistent)	149 (17.0%)	444 (10.1%)	96 (6.5%)	139 (5.8%)
CT (Lactase persistent)	326 (37.1%)	1722 (39.2%)	589 (39.5%)	922 (38.2%)
TT (Lactase persistent)	403 (45.9%)	2224 (50.7%)	803 (54.0%)	1355 (56.1%)
Cognitive test scores				
DWRT, mean (SD)	6.6 (1.5)	6.6 (1.5)	6.5 (1.5)	6.6 (1.5)
DSST, mean (SD)	42.6 (15.2)	44.6 (14.4)	45.2 (13.7)	45.6 (13.6)
WFT, mean (SD)	31.2 (12.9)	33.4 (12.4)	33.5 (12.6)	33.8 (12.3)

Abbreviations: BMI, body mass index; APOEe4, apolipoprotein epsilon 4 alleles; DWRT, delayed word recall test; DSST, digit symbol substitution test; WFT, word fluency test.

**Table 2:** Estimated, adjusted * race-specific difference in the 20-year change in cognitive performance by milk intake category. ARIC Study.

Test	20-Year Decline	Difference	Percent
Global z			
Almost never	−0.94 (−1.00, −0.88)	ref	ref
<1 glass/day	−0.99 (−1.01, −0.96)	−0.05 (−0.11, 0.02)	5%
1 glass/day	−1.00 (−1.05, −0.95)	−0.06 (−0.13, 0.02)	6%
>1 glass/day	−1.04 (−1.08, −1.01)	−0.10 (−0.16, −0.03)	11%
DWRT z			
Almost never	−1.15 (−1.23, −1.06)	ref	ref
<1 glass/day	−1.19 (−1.23, −1.15)	−0.04 (−0.13, 0.06)	3%
1 glass/day	−1.18 (−1.26, −1.11)	−0.03 (−0.14, 0.08)	3%
>1 glass/day	−1.25 (−1.31, −1.19)	−0.10 (−0.20, 0.00)	9%
DSST z			
Almost never	−0.78 (−0.82, −0.74)	ref	ref
<1 glass/day	−0.82 (−0.84, −0.80)	−0.04 (−0.09, 0.00)	5%
1 glass/day	−0.85 (−0.89, −0.81)	−0.07 (−0.12, −0.01)	9%
>1 glass/day	−0.87 (−0.89, −0.84)	−0.09 (−0.14, −0.03)	12%
WFT z			
Almost never	−0.24 (−0.29, −0.19)	ref	ref
<1 glass/day	−0.28 (−0.30, −0.26)	−0.04 (−0.09, 0.02)	16%
1 glass/day	−0.26 (−0.30, −0.22)	−0.02 (−0.08, 0.05)	8%
>1 glass/day	−0.29 (−0.33, −0.26)	−0.05 (−0.11, 0.01)	21%

Abbreviations: DWRT, delayed word recall test; DSST, digit symbol substitution test; WFT, word fluency test. Global z is a summary score, equal to the average of the three domain-specific z-scores. * Models adjusted for age, gender, race-center, education level, APOE4, BMI, smoking, alcohol intake, diabetes, physical activity, total energy intake, and diet quality. In column “Percent”, positive values represent % additional decline relative to the referent group.

## Supplementary Materials

The following are available online at www.mdpi.com/2072-6643/9/10/1134/s1, Table S1: Energy adjusted diet composition of study participants by milk intake group, mean (SE). ARIC Study, Table S2: Change in global *z* score by follow-up time period. ARIC Study, Table S3: Mean intake of milk and other dairy products by milk intake group. ARIC Study, Table S4: Distribution of milk intake groups and other dairy intake by total dairy intake quartiles. ARIC Study. Table S5. Estimated, adjusted * difference in the 20-year cognitive change by type of dairy intake. ARIC Study.
