# Supplementary material for: Milk Intake at Midlife and Cognitive Decline over 20 Years. The Atherosclerosis Risk in Communities (ARIC) Study
Source: Nutrients. 2017 Oct 17;9(10):1134. doi: 10.3390/nu9101134 (PMC5691750; doi:10.3390/nu9101134)
Supplement: Supplementary file 1 [file nutrients-09-01134-s001.zip › tables and figures.docx]

Milk Intake at Midlife and Cognitive Decline over 20 years. The Atherosclerosis Risk in Communities (ARIC) study.

*Natalia Petruski-Ivleva, Anna Kucharska-Newton, Priya Palta, David Couper, Lyn M. Steffen, Katie Meyer, Misa Graff, Bernhard Haring, A. Richey Sharrett, Gerardo Heiss.*

Tables and Figures

Figure 1. Timeline of the ARIC study.


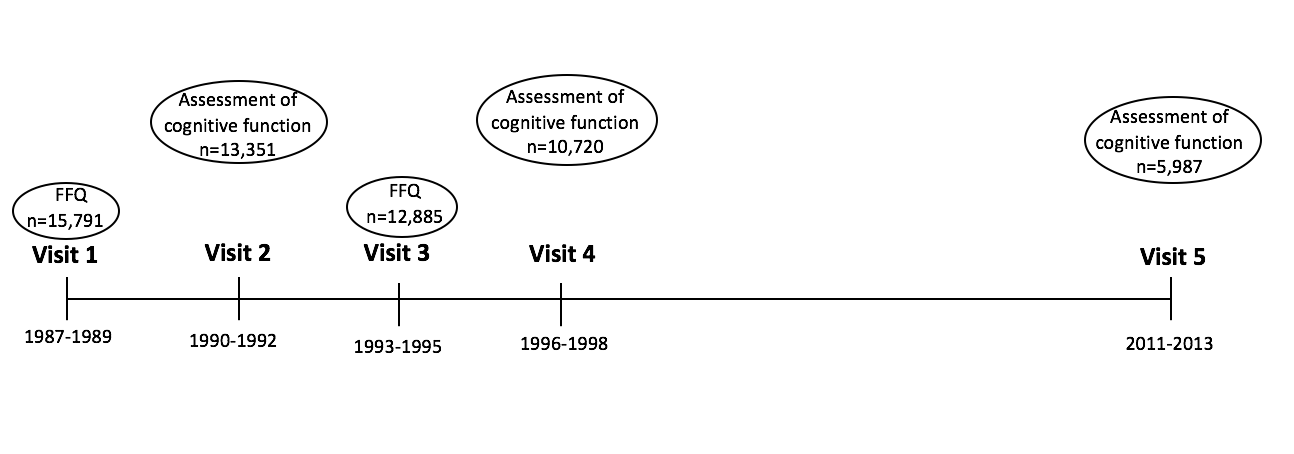


Table 1. Baseline (Visit 2) characteristics of study participants by milk intake group. ARIC Study, 1990-1992.

|  | Milk intake group | | | |
| --- | --- | --- | --- | --- |
|  | Almost Never | <1glass/day | 1glass/day | >1 glass/day |
|  | n=1554 | n=6872 | n=2036 | n=3290 |
| Age, mean (SD) | 56.7 (5.6) | 57.2 (5.6) | 58.5 (5.7) | 57.9 (5.8) |
| Black, % | 530 (34.1%) | 1833 (26.7%) | 360 (17.7%) | 542 (16.5%) |
| Female, % | 1023 (65.8%) | 3879 (56.4%) | 1096 (53.8%) | 1664 (50.6%) |
| Study site, %  Forsyth County, NC | 328 (21.1%) | 1894 (27.6%) | 584 (28.7%) | 760 (23.1%) |
| Jackson, MS | 484 (31.1%) | 1641 (23.9%) | 320 (15.7%) | 469 (14.3%) |
| Minneapolis, MN | 335 (21.6%) | 1499 (21.8%) | 601 (29.5%) | 1293 (39.3%) |
| Washington County, MD | 407 (26.2%) | 1838 (26.7%) | 531 (26.1%) | 768 (23.3%) |
| Education, % <High School | 415 (26.8%) | 1474 (21.5%) | 390 (19.2%) | 627 (19.1%) |
| Smoking, % Never | 564 (36.3%) | 2771 (40.3%) | 839 (41.2%) | 1301 (39.6%) |
| Drinking, % Never | 366 (23.6%) | 1582 (23.0%) | 476 (23.4%) | 654 (19.9%) |
| BMI (kg/m^2^), mean (SD) | 27.9 (5.7) | 28.1 (5.5) | 27.7 (5.1) | 27.9 (5.1) |
| Diabetes, % | 220 (14.3%) | 945 (13.8%) | 311 (15.3%) | 555 (16.9%) |
| Hypertension, % | 622 (40.2%) | 2451 (35.7%) | 721 (35.6%) | 1076 (32.8%) |
| Diet score, mean (SD) | 19.3 (4.9) | 20.7 (4.7) | 22.0 (4.7) | 22.1(4.8) |
| Lactose intake (g), mean (SD) | 2.3 (3.0) | 7.7 (5.5) | 14.9 (3.5) | 27.8 (15.4) |
| Physical activity (met-min/week) | 500 (647) | 674 (825) | 822 (907) | 728 (782) |
| APOEe4 allele, % present | 565 (33.8%) | 2218 (30.2%) | 669 (30.7%) | 1071 (30.3%) |
| Lactase persistence (Whites)  CC (Lactase non-persistent) | 149 (17.0%) | 444 (10.1%) | 96 (6.5%) | 139 (5.8%) |
| CT (Lactase persistent) | 326 (37.1%) | 1,722 (39.2%) | 589 (39.5%) | 922 (38.2%) |
| TT (Lactase persistent) | 403 (45.9%) | 2,224 (50.7%) | 803 (54.0%) | 1,355 (56.1%) |
| Cognitive test scores |  |  |  |  |
| DWRT, mean (SD) | 6.6 (1.5) | 6.6 (1.5) | 6.5 (1.5) | 6.6 (1.5) |
| DSST, mean (SD) | 42.6 (15.2) | 44.6 (14.4) | 45.2 (13.7) | 45.6 (13.6) |
| WFT, mean (SD) | 31.2 (12.9) | 33.4 (12.4) | 33.5 (12.6) | 33.8 (12.3) |

Abbreviations: BMI, body mass index; APOEe4, apolipoprotein epsilon 4 alleles; DWRT, delayed word recall test; DSST, digit symbol substitution test; WFT, word fluency test.

Figure 2. Estimated, adjusted* difference in the 20-year change in cognitive performance by milk intake group relative to those who reported “almost never” consuming milk. ARIC Study.


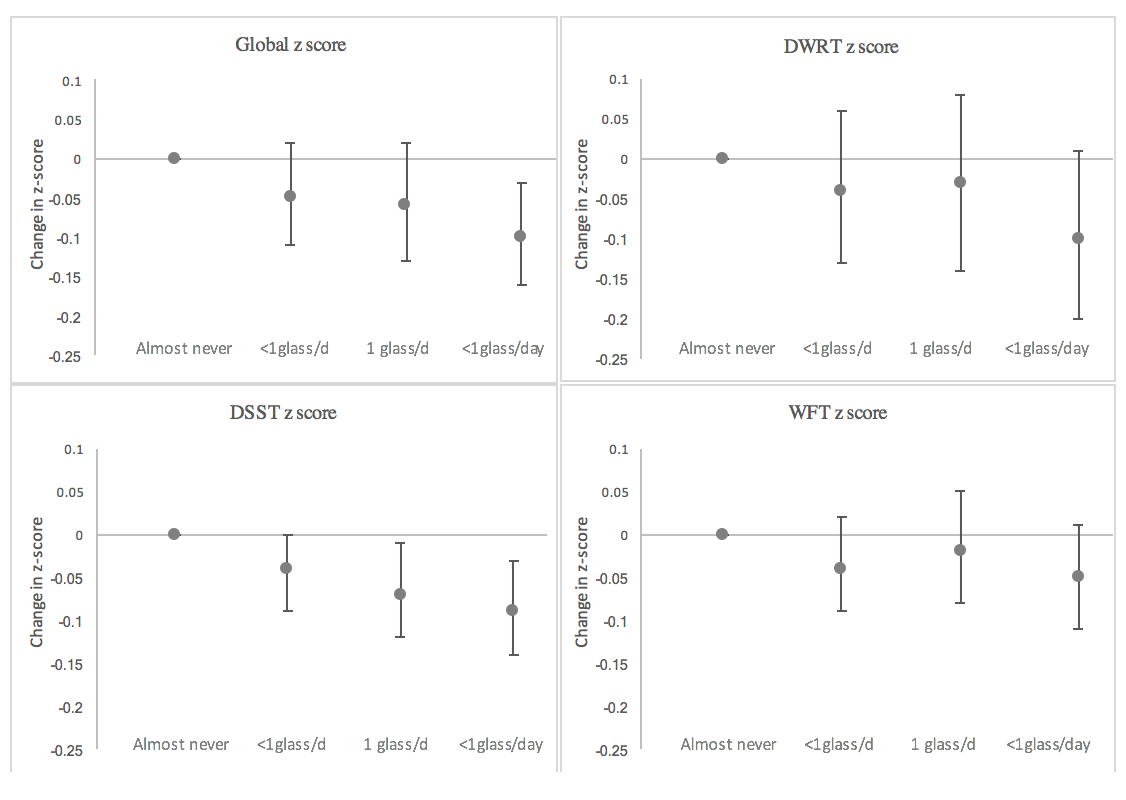


Abbreviations: DWRT, delayed word recall test; DSST, digit symbol substitution test; WFT, word fluency test.

Global z is a summary score, equal to the average of the three domain-specific z-scores.

* Estimates from models adjusted for age, gender, race-center, education level, APOE4, BMI, smoking, alcohol intake, diabetes, physical activity, total energy intake and diet quality score.

Figure 3. Estimated, adjusted* difference in the 20-year change in global-z score stratified by race and by LP/LNP genotype among Whites. ARIC Study.


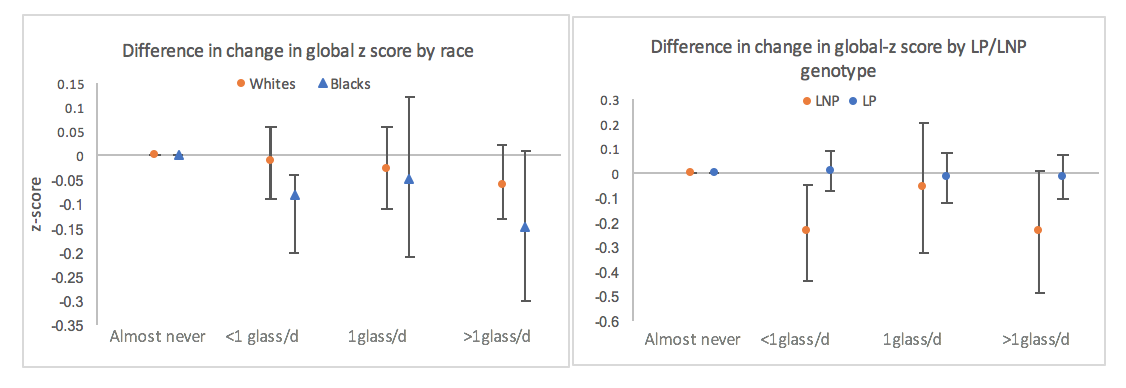


Abbreviations: LNP, lactase non-persistence; LP, lactase persistence.

* Models adjusted for age, gender, race-center, education level, APOE4, BMI, smoking, alcohol intake, diabetes, physical activity, total energy intake and diet quality. “Almost never” used as a referent category.

| Table 2. Estimated, adjusted* race-specific difference in the 20-year change in cognitive performance by milk intake category. ARIC Study. | | | |
| --- | --- | --- | --- |
| Test |  |  |  |
|  | 20-year decline | Difference | Percent |
| Global z |  |  |  |
| Almost never | -0.94 (-1.00, -0.88) | ref | ref |
| <1 glass/d | -0.99 (-1.01, -0.96) | -0.05 (-0.11, 0.02) | 5% |
| 1 glass/d | -1.00 (-1.05, -0.95) | -0.06 (-0.13, 0.02) | 6% |
| >1 glass/d | -1.04 (-1.08, -1.01) | **-0.10 (-0.16, -0.03)** | **11%** |
| DWRT z |  |  |  |
| Almost never | -1.15 (-1.23, -1.06) | ref | ref |
| <1 glass/d | -1.19 (-1.23, -1.15) | -0.04 (-0.13, 0.06) | 3% |
| 1 glass/d | -1.18 (-1.26, -1.11) | -0.03 (-0.14, 0.08) | 3% |
| >1 glass/d | -1.25 (-1.31, -1.19) | **-0.10 (-0.20, 0.00)** | **9%** |
| DSST z |  |  |  |
| Almost never | -0.78 (-0.82, -0.74) | ref | ref |
| <1 glass/d | -0.82 (-0.84, -0.80) | **-0.04 (-0.09, 0.00)** | **5%** |
| 1 glass/d | -0.85 (-0.89, -0.81) | **-0.07 (-0.12, -0.01)** | **9%** |
| >1 glass/d | -0.87 (-0.89, -0.84) | **-0.09 (-0.14, -0.03)** | **12%** |
| WFT z |  |  |  |
| Almost never | -0.24 (-0.29, -0.19) | ref | ref |
| <1 glass/d | -0.28 (-0.30, -0.26) | -0.04 (-0.09, 0.02) | 16% |
| 1 glass/d | -0.26 (-0.30, -0.22) | -0.02 (-0.08, 0.05) | 8% |
| >1 glass/d | -0.29 (-0.33, -0.26) | -0.05 (-0.11, 0.01) | 21% |

Abbreviations: DWRT, delayed word recall test; DSST, digit symbol substitution test; WFT, word fluency test.

Global z is a summary score, equal to the average of the three domain-specific z-scores.

* Models adjusted for age, gender, race-center, education level, APOE4, BMI, smoking, alcohol intake, diabetes, physical activity, total energy intake and diet quality. In column “Percent” positive values represent % additional decline relative to the referent group.

Supplemental tables and figures

Table S1. Energy adjusted diet composition of study participants by milk intake group, mean (SE). ARIC Study.

|  | Milk intake category | | | |
| --- | --- | --- | --- | --- |
|  | Almost never | <1 glass/day | 1 glass/day | >1 glass/day |
| Protein (g) | 67.0 (0.4) | 71.0 (0.2) | 73.1 (0.3) | 78.1 (0.3) |
| Animal Protein (g) | 49.3 (0.4) | 53.0 (0.2) | 54.8 (0.3) | 61.0 (0.3) |
| Total Fat (g) | 59.1 (0.3) | 59.3 (0.1) | 57.3 (0.2) | 57.5 (0.2) |
| Animal Fat (g) | 35.3 (0.3) | 36.1 (0.1) | 35.0 (0.2) | 37.1 (0.2) |
| Carbs (g) | 199 (0.9) | 197 (0.4) | 203 (0.8) | 198 (0.6) |
| Fiber (g) | 16.6 (0.1) | 17.4 (0.1) | 18.5 (0.1) | 17.1 (0.1) |
| Omega3s (g) | 0.25 (0.01) | 0.27 (0.00) | 0.26 (0.00) | 0.24 (0.00) |
| Fruits (serv) | 1.41 (0.03) | 1.54 (0.01) | 1.78 (0.03) | 1.75 (0.02) |
| Vegetables (serv) | 1.71 (0.03) | 1.77 (0.01) | 1.77 (0.02) | 1.72 (0.02) |
| Whole grain (serv) | 0.68 (0.02) | 0.73 (0.01) | 0.88 (0.02) | 0.89 (0.01) |
| Fish (serv) | 0.32 (0.01) | 0.32 (0.00) | 0.31(0.01) | 0.29 (0.01) |
| Meat (serv) | 1.59 (0.02) | 1.51 (0.01) | 1.36 (0.01) | 1.28 (0.01) |
| Diet soda (8oz serv) | 0.53 (0.02) | 0.54 (0.01) | 0.54 (0.02) | 0.51 (0.02) |
| SSB (8oz serv) | 0.82 (0.02) | 0.58 (0.01) | 0.45 (0.02) | 0.35 (0.01) |
| Coffee and tea (8oz serv) | 2.49 (0.06) | 2.42 (0.03) | 2.33 (0.05) | 2.30 (0.04) |
| Total energy (kcal) | 1459 (13) | 1529 (6) | 1625 (11) | 1866 (10) |

Abbreviations: SSB, sugar sweetened beverages.

Meat includes combined poultry, processed meat, beef, pork, and lamb.

Table S2. Change in global z score by follow-up time period. ARIC Study.

| Milk intake group | (Visit 2 – Visit 4) | |
| --- | --- | --- |
|  | Decline | Difference |
| Almost never | -0.12 (-0.15, -0.09) | Ref |
| <1glass/day | -0.12 (-0.14, -0.11) | -0.00 (-0.04, 0.03) |
| 1 glass/day | -0.11 (-0.14, -0.08) | 0.01(-0.03, 0.05) |
| >1glass/day | -0.16 (-0.18, -0.14) | -0.04 (-0.08, -0.01) |
|  | (Visit 4 – Visit 5) | |
| Almost never | -0.80 (-0.85, -0.76) | Ref |
| <1glass/day | -0.85 (-0.87, -0.83) | -0.05 (-0.10, -0.00) |
| 1 glass/day | -0.89 (-0.93, -0.85) | -0.09 (-0.15, -0.03) |
| >1glass/day | -0.86 (-0.89, -0.83) | -0.06 (-0.11, -0.01) |
|  | (Visit 2 – Visit5) | |
| Almost never | -0.92 (-0.98, -0.87) | Ref |
| <1glass/day | -0.97 (-1.00, -0.87) | -0.05 (-0.11, 0.01) |
| 1 glass/day | -1.00 (-1.04, -0.95) | -0.08 (-0.15, -0.01) |
| >1glass/day | -1.02 (-1.05, -0.99) | -0.10 (-0.16, -0.01) |

Global z is a summary score, equal to the average of the three domain-specific z-scores.

Table S3. Mean intake of milk and other dairy products by milk intake group. ARIC Study.

| Milk intake group | Total milk (glasses/day) | Skim milk  (glasses/day) | Whole milk  (glasses/day) | All dairy*  (servings/day) | Dairy other than milk **  (sevings/day) |
| --- | --- | --- | --- | --- | --- |
| Almost never | 0 | 0 | 0 | 0.61 (0.59) | 0.61 |
| <1 glass/day | 0.44 (0.26) | 0.33 (0.28) | 0.11 (0.19) | 1.15 (0.67) | 0.71 |
| 1 glass/day | 1.00 (0) | 0.82 (0.35) | 0.18 (0.35) | 1.80 (0.65) | 0.80 |
| >1 glass/day | 2.08 (0.9) | 1.68 (1.04) | 0.41 (0.78) | 3.00 (1.23) | 0.92 |

*All dairy=skim/low fat milk + whole milk +yogurt + ice-cream + cottage cheese + other cheese + butter

**Dairy other than milk= yogurt + ice-cream + cottage cheese + other cheese + butter

Table S4. Distribution of milk intake groups and other dairy intake by total dairy intake quartiles. ARIC Study.

|  | Total dairy intake quartile | | | |
| --- | --- | --- | --- | --- |
|  | 1 | 2 | 3 | 4 |
| Milk group |  |  |  |  |
| Almost never | 32.8% | 7.9% | 2.7% | 1.1% |
| <1 glass/day | 67.2% | 73.7% | 45.3% | 13.5% |
| 1 glass/day | 0 | 16.3% | 30.8% | 12.4% |
| >1 glass/day | 0 | 2.0% | 21.1% | 73.0% |
| Total milk intake (glass/day), mean (SD) | 0.17 (0.19) | 0.55 (0.33) | 0.88 (0.41) | 1.89 (1.06) |
| All-dairy intake (serving/day), mean (SD) | 0.48 (0.23) | 1.11 (0.15) | 1.72 (0.22) | 3.23 (1.11) |

Table 5S. Estimated, adjusted* difference in the 20-year cognitive change by type of dairy intake. ARIC Study.

|  | Whites | | | |
| --- | --- | --- | --- | --- |
|  | Global z score | DWRT z score | DSST z score | WFT z score |
| Total milk |  |  |  |  |
| Almost never | ref | ref | ref | ref |
| <1 glass/day | -0.01 (-0.09, 0.06) | -0.02 (-0.13, 0.09) | -0.00 (-0.05, 0.05) | -0.02 (-0.08, 0.05) |
| 1 glass/day | -0.03 (-0.11, 0.06) | -0.04 (-0.17, 0.09) | -0.02 (-0.08, 0.04) | -0.01 (-0.09, 0.06) |
| >1 glass/day | -0.06 (-0.13, 0.02) | -0.08 (-0.20, 0.04) | 0.00 (-0.05, 0.06) | -0.04 (-0.11, 0.03) |
| Skim milk |  |  |  |  |
| Almost never | ref | ref | ref | ref |
| <1 glass/day | -0.02 (-0.07, 0.04) | -0.01 (-0.10, 0.08) | -0.03 (-0.07, 0.01) | -0.00 (-0.05, 0.05) |
| 1 glass/day | -0.06 (-0.13, 0.01) | -0.08 (-0.18, 0.03) | -0.04 (-0.09, 0.11) | -0.01 (-0.08, 0.05) |
| >1 glass/day | -0.08 (-0.15, -0.01) | -0.10 (-0.10, 0.01) | -0.05 (-0.10, 0.00) | -0.04 (-0.10, 0.03) |
| Total dairy |  |  |  |  |
| 1^st^ quartile | ref | ref | ref | ref |
| 2^nd^ quartile | 0.01 (-0.05, 0.07) | -0.02 (-0.11, 0.08) | 0.00 (-0.04, 0.05) | 0.03 (-0.02, 0.09) |
| 3^rd^ quartile | 0.00 (-0.06, 0.06) | 0.01 (-0.08, 0.10) | 0.00 (-0.04, 0.04) | -0.00 (-0.06, 0.05) |
| 4^th^ quartile | -0.06 (-0.12, 0.00) | -0.09 (-0.18, -0.00) | -0.01 (-0.05, 0.03) | -0.02 (-0.07, 0.03) |

|  | Blacks | | | |
| --- | --- | --- | --- | --- |
|  | Global z score | DWRT z score | DSST z score | WFT z score |
| Total milk |  |  |  |  |
| Almost never | ref | ref | ref | ref |
| <1 glass/day | -0.08 (-0.20, -0.04) | -0.07 (-0.25, 0.12) | -0.06 (-0.15, 0.03) | -0.09 (-0.19, 0.00) |
| 1 glass/day | -0.05 (-0.21, 0.12) | 0.06 (-0.20, 0.32) | -0.02 (-0.15, 0.10) | -0.06 (-0.20, 0.07) |
| >1 glass/day | -0.15 (-0.30, 0.01) | -0.11 (-0.34, 0.12) | -0.10 (-0.21, 0.01) | -0.11 (-0.23, 0.01) |
| Skim milk |  |  |  |  |
| Almost never | ref | ref | ref | ref |
| <1 glass/day | -0.04 (-0.13, 0.05) | -0.05 (-0.19, 0.09) | -0.03 (-0.10, 0.04) | -0.04 (-0.12, 0.03) |
| 1 glass/day | -0.04 (-0.22, 0.13) | -0.10 (-0.17, 0.37) | -0.06 (-0.19, 0.08) | -0.06 (-0.21, 0.08) |
| >1 glass/day | -0.27 (-0.46, 0.10) | -0.25 (-0.53, 0.03) | -0.21 (-0.35, -0.07) | -0.10 (-0.25, 0.04) |
| Total dairy |  |  |  |  |
| 1^st^ quartile | ref | ref | ref | ref |
| 2^nd^ quartile | 0.06 (-0.04, 0.17) | 0.11 (-0.06, 0.27) | 0.03 (-0.06,0.11) | 0.01 (-0.08, 0.09) |
| 3^rd^ quartile | 0.00 (-0.11, 0.11) | 0.05 (-0.13, 0.23) | -0.03 (-0.12, 0.06) | -0.00 (-0.10, 0.09) |
| 4^th^ quartile | -0.07 (-0.20, 0.06) | 0.03 (-0.18, 0.24) | -0.12 (-0.22, -0.02) | -0.06 (-0.17, 0.05) |

Abbreviations: DWRT, delayed word recall test; DSST, digit symbol substitution test; WFT, word fluency test.

Global z is a summary score, equal to the average of the three domain-specific z-scores.

* Model adjusted for age, gender, race-center, education level, APOE4, BMI, smoking, alcohol intake, diabetes, physical activity, total energy intake and diet quality.
